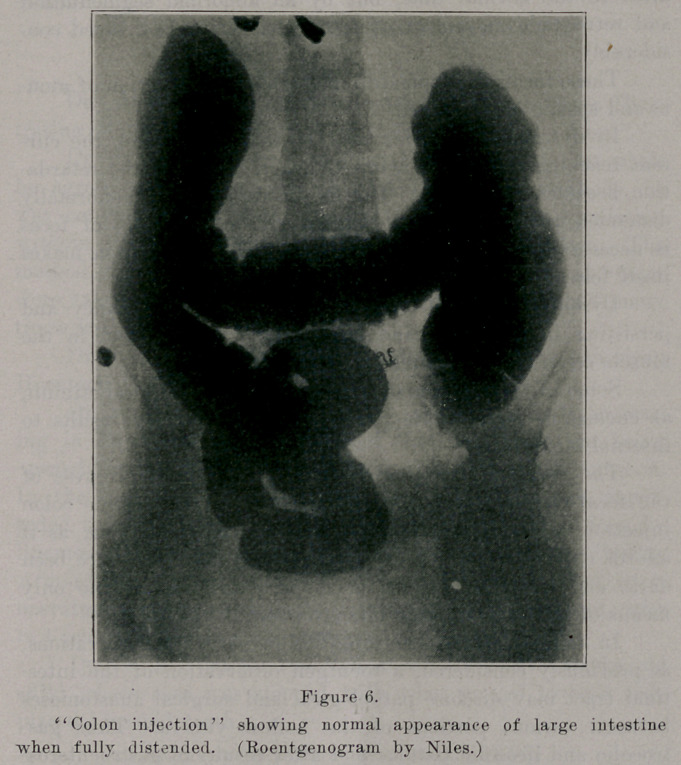# A Study in Intestinal Peristalsis

**Published:** 1914-10

**Authors:** George M. Niles

**Affiliations:** Professor of Gastro-Enterology and Clinical Medicine, Atlanta Medical College, Atlanta, Ga.


### A STUDY IN INTESTINAL PERISTALSIS

George M. Niles, M. D., Professor of Gastro-Enterology and Clinical Medicine, Atlanta Medical College, Atlanta, Ga.

This is a profitable subject for study and discussion. The progress of the intestinal current, its ebb and flow, the various vicissitudes in its abdominal journey, the impediments to its uninterrupted passage, the chemical and bacterial transmutations, the variation in reaction, and the transition from a normally acid chyme into a fecal mass—all are of the utmost interest to the thoughtful student.

The investigation of intestinal peristalsis has been more thorough and satisfactory by far since the roentgen ray has become available, and the following statements and conclusions are based upon visual observations and roentgenograms by myself and other laborers in this fruitful field.

Figure 1.

Coloptosis with acute angulation at splenic flexure. In spite of displacement, no clinical symptoms of ptosis or kinks were manifest. Upon operation a chronically diseased appendix was removed. (Roentgenogram by Niles.)

The opaque meal is the point of departure from ordinary methods of examination, for the hollow viscera are practically invisible unless a contrast is produced by artificial means between them and the surrounding structures. This contrast has heretofore in the main been produced by the introduction of large quantities of bismuth into the food or some suitable vehicle, but at present barium sulphate is being used principally. The latter agent, besides being less toxic and exerting less inhibitory effect on peristaltic progress, is also considerably cheaper than bismuth.

After the opaque meal leaves the stomach, with the exception of a small amount in the bulbus duodeni, it cannot be followed with ease during its passage through the small intestine, as the rate is so rapid. About the most that can be noted are small flecks scattered throughout the duodenum, jejunum and ileum, these being neither uniform nor enlightening.

The first part of the duodenum, or the bulbus duodeni, retains the barium because of its different anatomical structure. Instead of the valvulae conniventes found in other portions of the duodenum, the surface is perfectly smooth. Physiologically the bulbus duodeni is quite important, as it receives the acid chyme from the stomach, neutralizing it before it passes through the intestinal tract beyond. This portion of the duodenum may be said to "bear the brunt" of the first onrush of acid stomach contents, and for this reason we find the greater number of duodenal lesions near the pyloric orifice. Normally the barium meal should pass through the entire duodenum in from twenty-five to sixty seconds.

There may be ptoses, or kinks or stenoses of the duodenum, either or all tending to retard normal intestinal peristalsis.

The jejunum and ileum, forming the remaining portions of the small intestine, propel their contents rapidly. Like the duodenum they possess both mixing and onward peristaltic waves, though these waves are very slight in the lower part of the ileum, where the chyme collects before entering the cecum. Should there be ileo-cecal insufficiency, or incompetency of the ileo-cecal valve, there may be demonstrated much reverse peristalsis here, with tumultuous movements almost amounting to spasm during certain periods of progress.

Some portions of the opaque meal should appear in the cecum four hours after its entrance into the duodenum, while much of it should be collected in the lower ileum after that time. After six hours only a part should remain in the ileum, and any considerable residue remaining after eight hours would be pathologic. Normally there is a tonic contraction at the ileo-cecal valve, giving rise to an apparent damming just at that portion of the small gut.

Figure 2.

Roentgenogram taken eight hours after barium meal. Hurried peristalsis is shown, as meal has left transverse colon, while some has already passed through the rectum. (Roentgenogram by Niles.)

A number of factors may abnormally influence peristalsis in the small intestine, as bands due to adhesions following inflammatory processes, strictures of the lumen, growths within the gut or external to it, displacements, kinks or ptoses. In studying the progress and emptying time of the ileum, however, due allowance must be made for delay or otherwise in the evacuation of the stomach, as this would naturally modify the advance of the intestinal current.

Marked interruption, where the passage of the chyme into the cecum is delayed as much as 24, 48 or even 72 hours, generally denotes material obstructions. Kinks and ptoses seldom delay this passage more than 12 hours, and rarely call for surgical interference.

Leaving the consideration of the small intestine, we follow the onward current into the large intestine, which is divided into the cecum, ascending colon, transverse colon, sigmoid flexure and rectum.

A slight mention of the anatomical relation of these various portions will assist in appreciating pathologic variation in form and function. The cecum is that portion of the large intestine that lies below the ileo-cecal valve. It is generally freely movable, and may be markedly so because of its mesenteric attachment. It may descend into the true pelvis or be capable of considerable lateral displacement without producing any pathologic conditions or distressing symptoms. Those who consider a "cecum mobile" a pathologic entity—one to be attacked surgically, might with profit read the notable battle of that redoubtable Don Quixotte and his charge upon the windmills.

The ascending colon extends upward, backward into the iliac fossa, reaching nearly to the liver, where it forms a more or less acute angle with the transverse colon, called the hepatic flexure. This flexure, resting obliquely backward in the right iliac fossa, is not displaced downward readily, though this occurs at times. As this is the largest part of the colon in diameter, it often remains filled for quite a long time. The hepatic flexure with the first portion of the ransverse colon may be markedly ptosed without producing any symptoms of obstruction.

The transverse colon, extending from the hepatic to the splenic flexure, is not in the horseshoe-shaped form as we have been taught. It follows an oblique direction, from below up-

Figure 3.

Roentgenogram showing "Dyschesia," or difficult defecation. Note collection of feces in ampulla of rectum. This was taken forty-eight hours after ingestion of opaque meal. (Roentgenogram by Niles.)

ward, along the greater curvature of the stomach, and may form a deep loop down even into the pelvis without causing abnormal symptoms. The latter third of this portion rises almost perpendicularly to the splenic flexure, there forming an acute angle with the descending colon. The splenic flexure, unlike the hepatic, is firmly held up to the diaphragm by a strong ileocolic ligament. The upper part of this flexure, when not actually filled with feces, is usually filled with gas, and the acuteness of the angle constitutes a natural hindrance to onward and easy peristalsis.

The descending colon extends forward and downward into the left iliac fossa, normally extending from the splenic flexure to the brim of the true pelvis.

The sigmoid flexure is variable in shape, length and position, attached as it is to a mesentery of variable length.

In all its parts the colon shows a haustral segmentation, less marked in the descending colon and sigmoid flexure. In spastic states of the large intestine, this segmentation is much more apparent.

The rectum or ampulla recti is the most distensible portion of this intestine, forming a reservoir for the feces. It seldom exhibits movements except during the act of defecation.

The distribution of the musculature of the large intestine gives rise to various forms of motion which can be studied roentgenologically.

These forms produce regular peristaltic movements, greater peristaltic movements, and storm-like movements occurring infrequently, but exerting marked displacement of the intestinal content toward the rectum. The lesser peristaltic movements, resulting from the contraction and relaxation of the circular musculature in a given area, yield but slight propulsive effect. These movements seem mostly mixing and segmenting in their character.

The greater peristaltic or wave movements extend over a larger area, showing more onward displacement of the feces. These, however, under certain circumstances, may be reversed.

Antiperistaltic movements have been demonstrated in the normal intestine, permitting a backward displacement of the fecal current.

Excessive longitudinal peristalsis or storm-like movements occur only about four to six times during the passage of the current through the colon, and can best be noted just before or during defecation. Visual observation at this time shows a sudden displacement of rather large masses of feces toward the anus.

Figure 4.

Fairly normal appearance of fecal current after twelve hours. Slight spasticity shown. (Roentgenogram by Niles.)

In conditions of hurried peristalsis, resulting from any cause, the barium meal may be noted in the splenic flexure and descending colon in less than four hours after its entrance into the small intestine.

The cecum and ascending colon, as the reservoir in which the cellulose undergoes bacterial action, retains the chyme much longer than other portions of the large intestine, and in constipated conditions may become greatly dilated.

Tuberculosis of the large intestine and ileocecal tuberculosis more frequently than tumors are the cause of intestinal

Figure 5.

Roentgenogram showing Dyskinetic, or spastic, form of constipation. This condition is aggravated by ordinary cathartics, but relieved by a liberal diet, petroleum oil and atropine. (Roentgenogram by Niles.)

stenosis. This condition should be suspected when the barium meal does not reach the cecum and ascending colon in four or five hours after leaving the stomach.

While ptoses and acute kinks undoubtedly exert an influence obstructive to normal peristalsis, we must nevertheless admit that we not infrequently find decided ptoses and apparently acute kinks in the intestinal tract of some individuals who report no clinical symptoms of intestinal stasis.

Constipation may be denominated as a condition, and this condition has been greatly elucidated by roentgenologic studies.

Figure 6.

"Colon injection" showing normal appearance of large intestine when fully distended. (Roentgenogram by Niles.)

The latest and seemingly most logical classification of constipation lies in the hypokinetic and dyskinetic forms, and dyschesia, or difficult defecation.

In the hypokinetic form there is lacking both muscular tone and motor stimuli. This motor deficiency may occur at any part of the colon, and can be located by an unduly prominent mass of feces there.

In the dyskinetic form, there is an excess of motility with marked antiperistalsis. The lower colon and rectum may be filled in the normal time, but by an abnormal segmentation and retrograde movement of feces, evacuation is delayed considerably.

These forms correspond to the former classification of atonic and spastic constipation.

In dyschesia the colon may be normally active, the current reaching the rectum without delay, but there the retardation becomes pathologic. This is the result of an abnormally distended ampulla recti, so that an excessive amount of feces is necessary before the requisite stimulus to defecation makes itself felt.

Dyschesia may be congenital, beginning in infancy and persisting in later life, and dependent upon a defect in the muscle sense of the rectum.

Some of these forms of dyschesia respond to local stimuli, as enemas or suppositories, but give unsatisfactory results to internal medication.

The roentgen examination of the downward progress of the fecal current or the upward progress of an opaque colon injection in the large intestine is of undisputed value, as it affords definite knowledge of a field which has heretofore been dark, and where an exploratory laparotomy seemed the only means of clearing up some obscure state.

In addition to the determination of peristaltic aberrations, as previously considered, a roentgen observation of the intestinal tract may disclose pathological and surgical anastomoses between various portions of the hollow viscera. Thus gastrocolic and ileocolic fistulae and after results of gastroenterostomies can with profit be studies by this method.

The subject of intestinal peristalsis is one too broad to be adequately covered in a brief paper. The lights and shadows as they appear under the fluoroscopic screen teach their own illuminating lesson, a lesson, which, if patiently studied and rightly interpreted, will speed our journey to the goal of correct apprehension of the physiology and pathology of this vital tract.

922 Candler Building.

## Figures and Tables

**Figure 1. f1:**
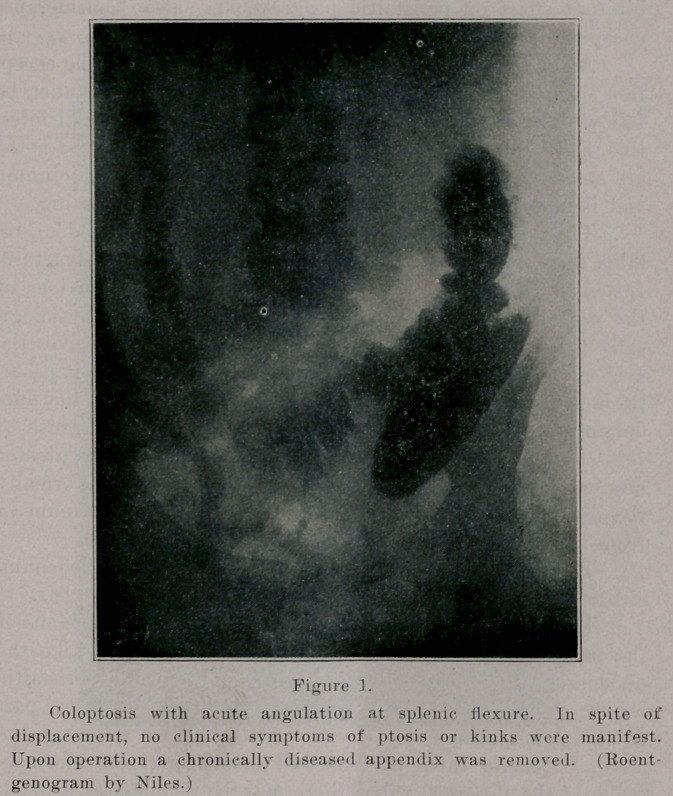


**Figure 2. f2:**
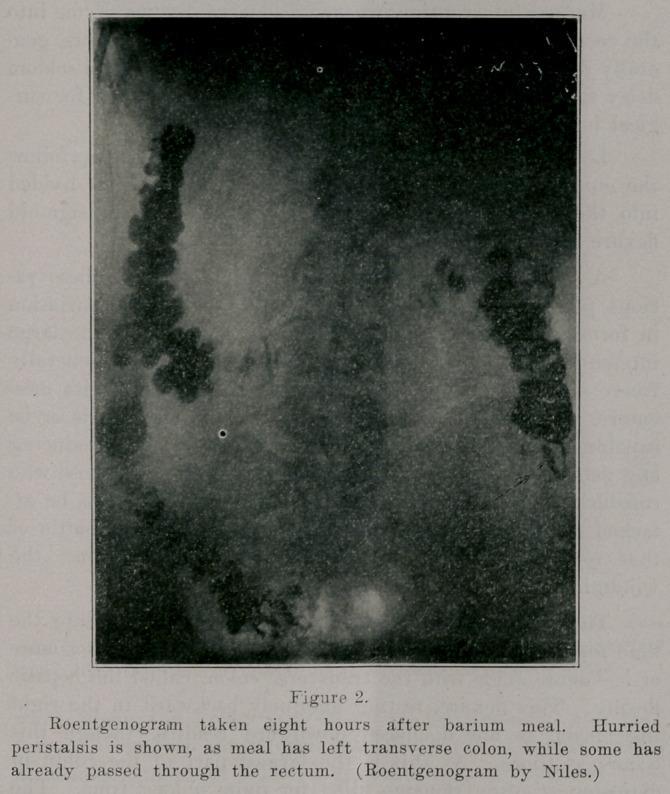


**Figure 3. f3:**
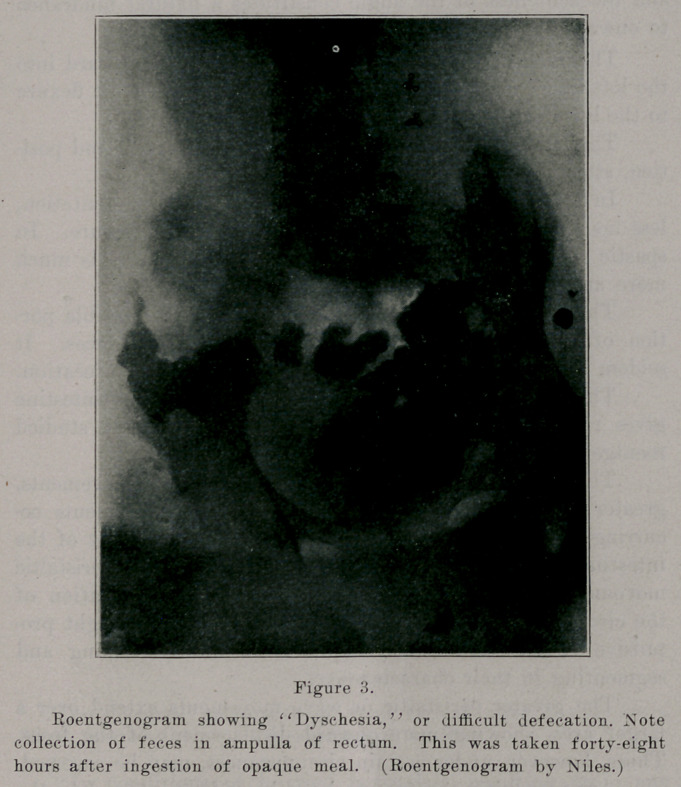


**Figure 4. f4:**
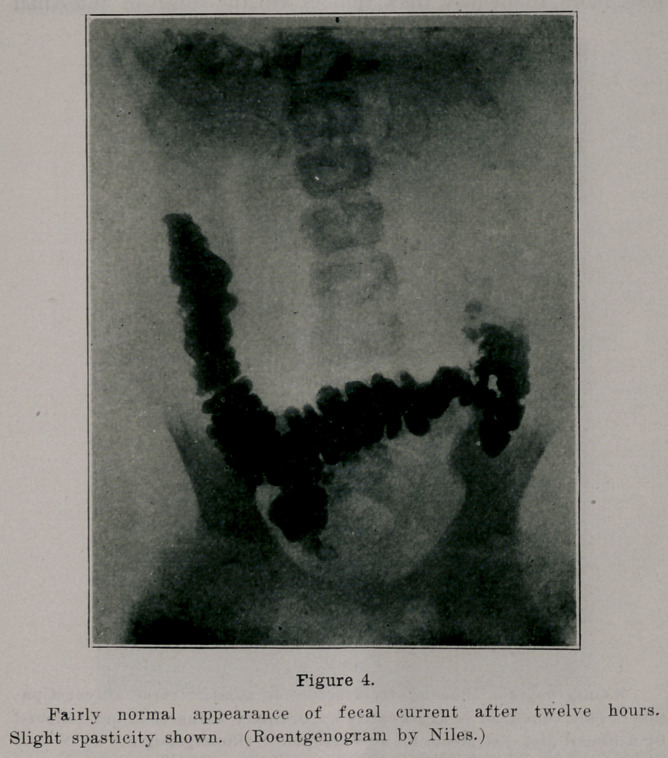


**Figure 5. f5:**
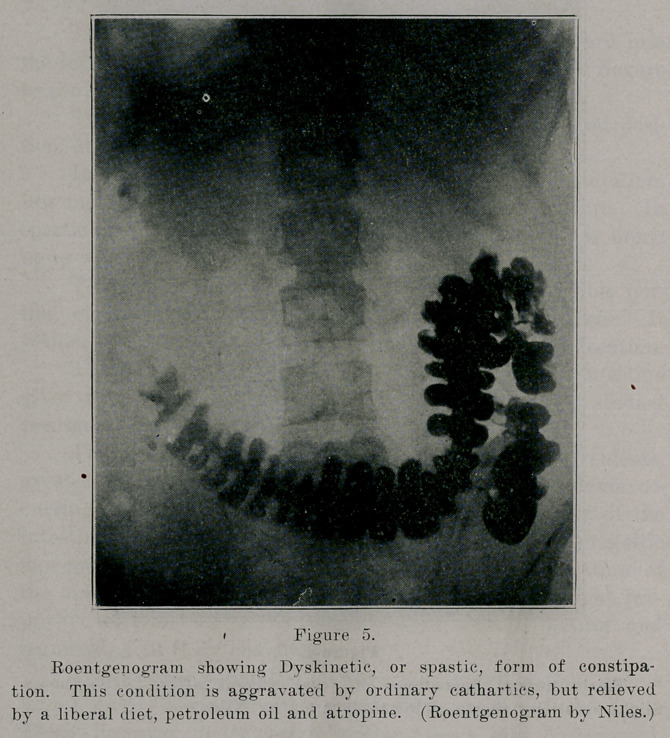


**Figure 6. f6:**